# The Antileukemia Activity of Natural Product HQ17(3) Is Possibly Associated with Downregulation of miR-17-92 Cluster

**DOI:** 10.1155/2014/306718

**Published:** 2014-07-22

**Authors:** Ya-Chun Liao, Tzu-Heng Lin, Chih-Ying Chen, Shwu-Bin Lin, Lo-Chun Au

**Affiliations:** ^1^Institute of Biotechnology in Medicine, Department of Biotechnology and Laboratory Science in Medicine, National Yang-Ming University, Taipei 11221, Taiwan; ^2^Department of Medical Research and Education, Taipei Veterans General Hospital, Taipei 11217, Taiwan; ^3^Department of Clinical Laboratory Sciences and Medical Biotechnology, National Taiwan University, Taipei 11217, Taiwan

## Abstract

The compound 10′(Z),13′(E),15′(E)-heptadecatrienylhydroquinone [HQ17(3)] was purified from the sap of the lacquer tree *Rhus succedanea*. HQ17(3) has cytotoxic effect on cancer cells and can inhibit topoisomerase (topo) II*α* activity. We treated various cancer cells with different doses of HQ17(3) and found that leukemia cells were most sensitive to HQ17(3). After analysis of microRNA (miRNA) profiling, we found that treatment with HQ17(3) caused downregulation of miR-17-92 cluster in some leukemia cells. These changes partially restored the normal levels from leukemia-specific miRNA expression signature. Messenger RNAs of tumor suppressor proteins, such as pRB, PTEN, and Dicer, are targets of miR-17-92 cluster. Their protein levels were increased after the treatment. c-Myc is a regulatory protein for miR-17-92 gene. Similar to topo II*α*, we found that c-Myc decreased its activity after the HQ17(3) treatment, which may explain the downregulation of miR-17-92 cluster. Combined with 5-fluorouracil, NaAsO_2_, or ABT-737, HQ17(3) elicited additive inhibitory effects on leukemia cells. In conclusion, the high sensitivity of leukemia cells to HQ17(3) may be associated with the reduction of topo II*α* and c-Myc activities, as well as with the downregulation of the miR-17-92 cluster expression.

## 1. Introduction

The compound 10′(Z),13′(E),15′(E)-heptadecatrienylhydroquinone [HQ17(3)] is a new natural product purified from the sap of the lacquer tree* Rhus succedanea* [1]. HQ17(3) is composed of 1.5% to 2% dry weight. HQ17(3) effectively and irreversibly inhibits topoisomerase (topo) II*α* activity by reacting with some cysteine residues of this enzyme [2]. A cell-based assay has shown that HQ17(3) inhibits the growth of topo II-deficient cells HL-60/MX2 with an EC50 of 9.6 *μ*M and exerts no effect on peripheral blood mononuclear cells at concentrations of up to 50 *μ*M [3]. Therefore, HQ17(3) attacks other targets on leukemia cells;, so obtaining more information about this drug is worthwhile.

MicroRNAs (miRNAs) are endogenous 21–24 nucleotide (nt) noncoding RNAs that function as posttranscriptional gene silencers for their target genes [4, 5]. Aberrant miRNA expression contributes to tumorigenesis and cancer progression [6, 7]. Aberrant expression of specific miRNAs has recently been discovered in chronic lymphocytic leukemia (CLL) and other B-cell lymphomas [8]. Polycistronic miR-17-92 cluster is also amplified and overexpressed in different types of B-cell lymphomas [9]. The miR-17-92 cluster contains miR-17, miR-18a, miR-19a, miR-20a, miR-19b, and miR-92 [10]. They are transcribed from an intron of the C13-25orf locus at 13q31-q32 amplification. c-Myc is a crucial upstream regulator of the miR-17-92 polycistron and is correlated with miR-17-92 levels [11, 12]. For BCR-ABL-positive cell lines, the oncogenic ABL variants induce expression of c-Myc [13].

The miR-17-92 polycistron promotes several aspects of oncogenic transformation, including evasion of apoptosis [14]. Further dissection of the miRNA components in this cluster reveals that the miR-17/20a seed family accounts for antisenescence activity, with targets such as pRB and E2F1 [15]. The miR-19a/b inhibits apoptosis by suppressing PTEN (a tumor suppressor protein) [16, 17]. The 3′UTR of Dicer messenger RNA (mRNA) contains two miR-18a binding sites. Dicer cleaves pre-miRNA to produce active miRNA duplex; it functions as an antiproliferation protein in general [18, 19]. Taken together, the members of miR-17-92 cluster promote tumorigenesis by antagonizing senescence, apoptosis, and tumor-suppressing mechanisms.

In this study, we found that treatment with HQ17(3) caused a decrease of c-Myc activity and downregulation of miR-17-92 clusters in some leukemia cells. This finding may explain why leukemia cells were selectively sensitive to this compound. HQ17(3) possibly has a significant role in antileukemia treatment.

## 2. Materials and Methods

### 2.1. HQ17(3)

The purification steps for HQ17(3) are the same as those described in our previous study [20]. HQ17(3) was dissolved in 50% alcohol and kept at −20°C. The working concentrations of HQ17(3) were 1, 3, and 9 mM. Therefore, cells treated with 1, 3, or 9 *μ*M of HQ17(3) can have the same concentration of alcohol. For blank control, the same amount of 50% alcohol was added.

### 2.2. Cell Viability

K562 (human myelogenous leukemia), U937 (human leukemic monocyte lymphoma), Molt-4 (human T lymphoblast cells), and Ramos (human B-cell lymphoma) were purchased from the Bioresource Collection and Research Center (Hsinchu, Taiwan). Cells were cultured in RPMI containing 10% fetal bovine serum, 1% nonessential amino acids (all from Gibco-BRL, Gaithersburg, MD, USA), 1% L-glutamine, and 1% sodium pyruvate (Sigma-Aldrich St. Louis, MO) in a humidified CO_2_ incubator at 37°C. Cells were seeded in a 12-well plate (4 × 10^5^ cells/well to 5 × 10^5^ cells/well) and treated with HQ17(3) and/or various concentrations of 5-fluorouracil (5-FU), ATB-737 (purchased from Selleckchem), or sodium arsenite (AsNaO_2_, dissolved in DMSO) for 24 h. After treatment, 100 *μ*L of CellQuanti-Blue (BioAssay System, Hayward, CA, USA) was added, and the cells were incubated for another 2 h. One hundred microliter of the cell lysate was withdrawn and subjected to assay of fluorescence (Ex 530 nm, Em 590 nm) by TECAN 1000 (Tecan Group, Ltd., Switzerland).

### 2.3. Microarray

The miRNAs from these cells were isolated by TRIzol (Invitrogen, Carlsbad, CA, USA) according to the protocol provided by the manufacturer. After passing the standard quality control, these miRNA levels were quantified by Agilent human miRNA array R12 and GeneSpring GX software. miRNA profiles were also measured by NanoString nCounter miRNA (NanoString Technologies, Inc., Seattle, WA, USA) which are based on direct digital detection of mRNA molecules using color-coded probes without the sequence amplification step [21].

### 2.4. Detection of miRNA Levels through qRT-PCR Analysis

The K562, Ramos, and Molt-4 cells were treated with 3 *μ*M of HQ17(3) for 24 h. Total RNA was extracted from the cells using TRIzol reagent (Invitrogen) according to the standard protocol provided by the manufacturer. The qRT-PCR analysis for miR-17, miR-19a, and U6 small nuclear RNA was performed using TaqMan MicroRNA assays kit (Applied Biosystem, Foster City, CA, USA) and under the conditions provided by Applied Biosystem. ΔCt was calculated by subtracting the Ct of U6 from that of miR-17 and miR-19a. ΔΔCt was calculated by subtracting the ΔCt of the untreated control from that of the HQ17(3)-treated sample. The fold changes in miR-17 and miR-19a levels were calculated as log⁡⁡2^−ΔΔCt^.

### 2.5. Detection of Protein Levels by Western Blot Analysis

HQ17(3)-treated cells in 6 cm dishes were rinsed twice with phosphate buffered saline (PBS). Two hundred microliters of RIPA lysis buffer (50 mM tris-HCl, pH 7.4, 150 mM NaCl, 1 mM EDTA, 1% Triton X-100, 1% sodium deoxycholate, 0.1% SDS) containing 2 *μ*L of 100x protease inhibitor complex (Calbiochem Nottingham, UK) was added. The cells were scraped from the Petri dish and transferred to an Eppendorf tube. The tube was placed on ice for 30 min with constant vortex. Cell lysate was centrifuged at 15,000 ×g for 30 min. Sixty micrograms of protein from the supernatant was loaded on 8% or 12% SDS-polyacrylamide gel, followed by western blot analysis. Anti-pRB antibody (Cat. number 9309P, Cell Signaling, Danvers, MA, USA), anti-Dicer (Cat. number ab14601, Abcam, Cambridge, UK), anti-PTEN (Cat. number 9552, Cell Signaling), c-Myc (Cat. number SC-40, Santa Cruz Biotechnology, Inc., Dallas, Texas), antibody against *β*-actin (Cat. number ACTB12-M, Alpha Diagnostic International, Inc., San Antonio, Texas, USA), and anti-mouse IgG secondary antibody conjugated with peroxidase (A9044, Sigma) were used in the western blot analysis. The immunoreactive bands were revealed by ECL system (Perkin Elmer, Inc., Boston, MA, USA) and developed on X-ray films.

### 2.6. c-Myc Activity Reporter Assay

Cignal c-Myc Reporter (FLuc) kit (SABioscience, Hilden, Germany) was used to detect c-Myc activity. Transient transfection was conducted. K562 cells at a density of 2 × 10^7^ cells in 0.4 mL of serum-free RPMI were loaded in a BTX electroporation cuvette (4 mm gap size). Then, 1 *μ*g Cignal c-Myc Reporter plasmid, positive or negative FLuc control, and 1 *μ*g RLuc (internal control) were added in the cuvette. Electroporation was conducted twice at 320 V for 35 ms using BTX ECM 830 machine (Harvard Apparatus, Holliston, MA, USA), followed by incubation at 37°C for 5 min. The cells were transferred into a 6 cm dish containing 5 mL of serum medium. HQ17(3) with the same volume of 50% EOH was added 1 h later and incubated for 24 h. Cells were harvested by centrifugation and washed twice with PBS; 250 *μ*L 1X Glo lysis buffer (Promega, Inc., Madison, WI, USA) was added and the mixture was left to stand for 5 min. The activities of FLuc and RLuc from the supernatants were measured using Bright-Glo Luciferase Assay System and Ready-To-Glow Reporter Assay (Clontech, Inc., Palo Alto, CA, USA), respectively, according to the manufacturer's instruction.

## 3. Results and Discussion

### 3.1. Cytotoxicity of HQ17(3) to Leukemia Cells

**Figure 1:**
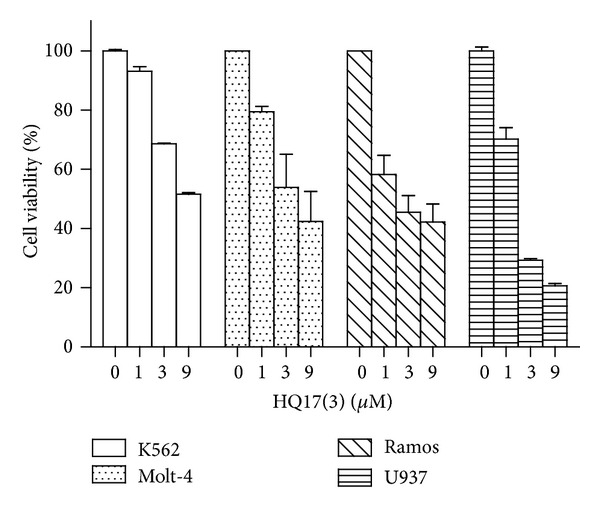
Viability for leukemia cells treated with HQ17(3). K562, Molt-4, Ramos, and U937 were treated with various doses of HQ17(3) for 24 h. The cells were then subjected to viability assay using a CellQuanti-Blue kit. The viability of blank is designated to be 100%. The experiments were conducted in triplicate. Means ± SD are shown.

Cell lines from different tissue origins were treated with various doses of HQ17(3). A549, H1299, HEK, HepG2, and MCF-7 were insensitive to HQ17(3). These cells maintained 80% viability after treatment with 9 *μ*M of HQ17(3) for 24 h (data not shown). However, leukemia cells were more sensitive to HQ17(3). K562, Molt-4, Ramos, and U937 were treated with various doses of HQ17(3) for 24 h. Their viabilities are shown in Figure 1.

### 3.2. Downregulation of miR-17-92 by HQ17(3)

**Figure 2:**
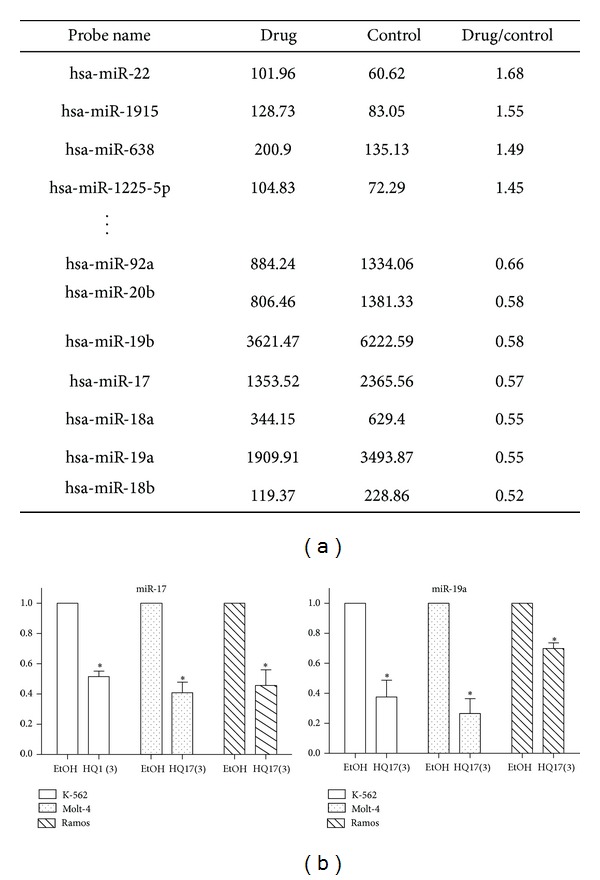
Detection of level change of miRNA upon treatment with HQ17(3). (a) U937 cells were cultured with/without 3 *μ*M of HQ17(3) for 24 h. The changes in miRNA levels were detected by an Agilent human miRNA array R12. The miRNA levels exhibiting apparent change (>1.43-fold or <0.7-fold and signal >60) were presented. (b) K562, Molt-4, and Ramos were cultured with/without 3 *μ*M of HQ17(3) for 24 h. The level change of miR-17 and miR-19 were monitored by qRT-PCR. The experiments were conducted in triplicate. Means ± SD are shown. *P < 0.05 compared to mock.

Aberrant expression of specific miRNAs has recently been addressed for CLL and other B-cell lymphomas [8]. Therefore, we monitored the change in miRNA profile after treatment with HQ17(3). U937 cells were treated with 3 *μ*M of HQ17(3) for 24 h. The changes in miRNA levels were detected by Agilent human miRNA array R12. The miRNA levels that showed apparent change (>1.43-fold or <0.7-fold and signal >60) were screened and presented in Figure 2(a). We found that the downregulated miRNAs, that is, miR-17, miR-18a, miR-19a/b, miR-20a, and miR-92a, all belong to a polycistron known as miR-17-92 cluster. We also measured miRNA profiles by NanoString nCounter miRNA. The treated Ramos cells (3 *μ*M, 24 h) showed different extents of downregulation of miR-17 (0.78), miR-18a (0.52), miR-19a (0.59), and miR-20a (0.92). Moreover, qRT-PCR was used to verify level change of miR-17 and miR-19a in K-562, Molt-4, and Ramos cells. The results are shown in Figure 2(b). Decrease in miR-17 and miR-19a was observed in these HQ17(3)-treated cells. We speculated that the downregulation of miRNA in miR-17-92 cluster after treatment with HQ17(3) may be a general phenomenon for leukemia cells.

### 3.3. Elevation of Tumor Suppressor by HQ17(3)

**Figure 3:**
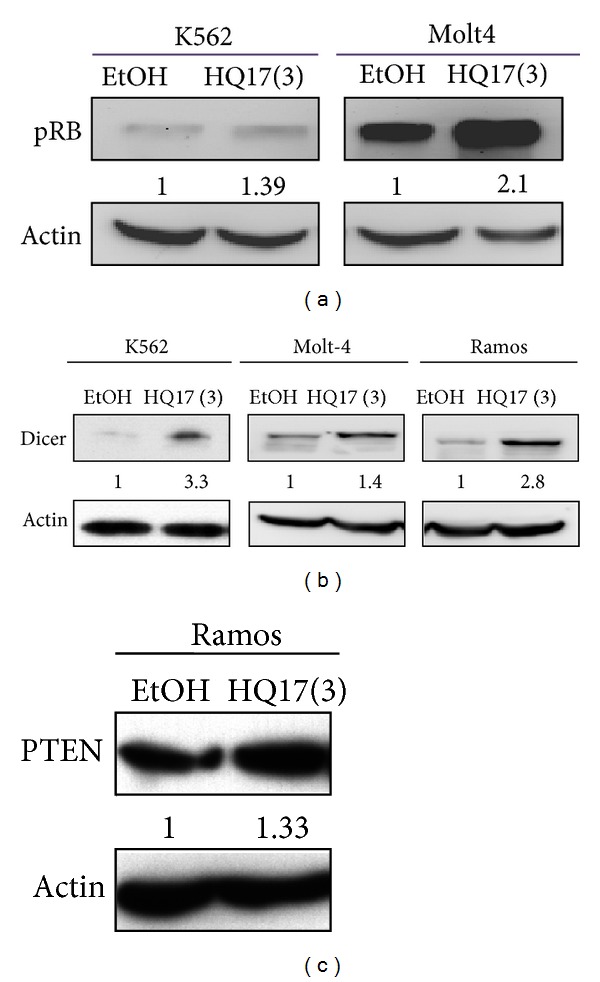
Detection of level change in tumor suppressor proteins upon treatment with HQ17(3). Leukemia cells were cultured with/without 3 *μ*M of HQ17(3) for 24 h. Western blot analyses were conducted to monitor pRB, PTEN, and Dicer of these cells. *β*-Actin was used as internal control for normalization.

Some mRNAs of tumor suppressor proteins, such as pRB, PTEN, and Dicer, are the targets for miRNA members of miR-17-92 cluster [15–17]. Therefore, the downregulation of miR-17-92 may cause an increase of these proteins. Western blot analyses were conducted. The results are shown in Figure 3. Protein levels of pRB, PTEN, and Dicer elevated to some extent for leukemia cells treated with HQ17(3).

### 3.4. The Role of c-Myc

**Figure 4:**
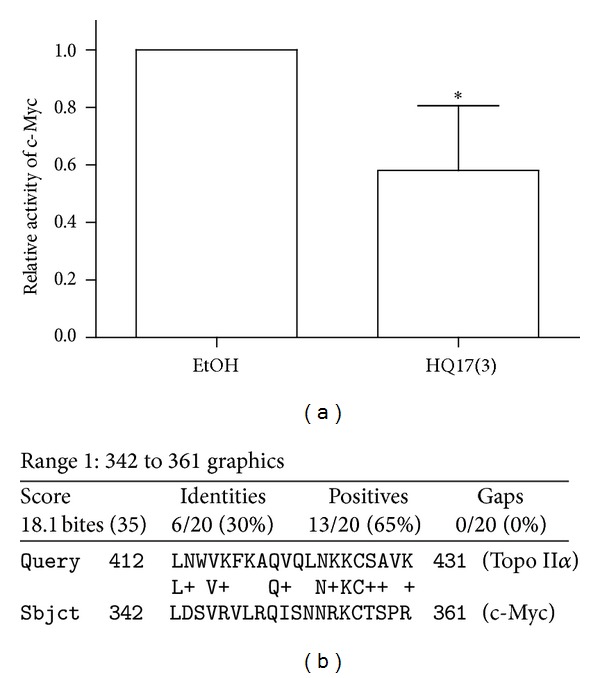
Monitoring of c-Myc activity upon treatment with HQ17(3) and putative reactive hot spot. (a) K562 cells were transfected with a c-Myc reporter with/without treatment with 3 *μ*M HQ17(3). c-Myc activity was monitored 24 h after the treatment. The experiments were repeated five times. Mean ± SD is shown. *P < 0.05 compared to mock. (b) Sequence homology was found when c-Myc and topo II*α* were aligned.

c-Myc is a crucial upstream regulator of the miR-17-92 polycistron. We wondered if the specific and general downregulation of miR-17-92 members are due to the partial inactivation of c-Myc by HQ17(3). K562 cells were transfected with c-Myc reporter with or without treatment of HQ17(3). The c-Myc activity was monitored within 24 h after the treatment. The result shown in Figure 4(a) indicates a significant decrease of c-Myc activity upon HQ17(3) treatment. Previous reports have indicated that HQ17(3) reacts and modifies some cysteine residues of topo II*α* in vitro and in cells [2]. However, Cys-427 modification was found only in the cellular system [2]. HQ17(3) also possibly reacts and modifies cysteine residues of c-Myc. Therefore, we created amino acid sequence alignments of c-Myc and topo II*α* using the website http://blast.ncbi.nlm.nih.gov/Blast.cgi?CMD=Web&PAGE_TYPE=BlastHome. Interestingly, a consensus-like sequence was found close to Cys-257 of c-Myc and Cys-427 of topo II*α* (Figure 4(b)). This consensus-like sequence may be a hot spot for the attack of HQ17(3).

### 3.5. Additive Effect of HQ17(3)

**Figure 5:**
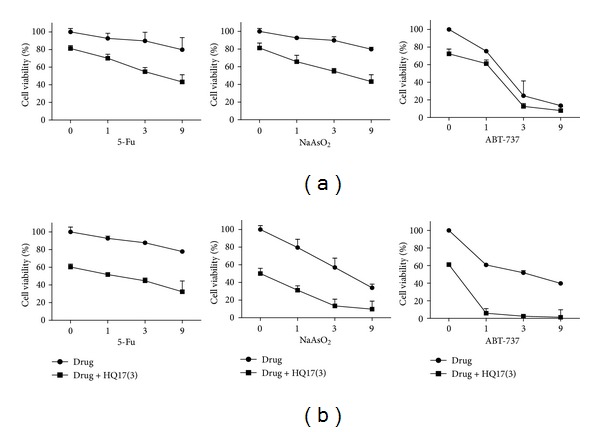
Effect of HQ17(3) in combination with anticancer drugs. Leukemia cell Molt (a) and Ramos (b) were treated with different doses of anticancer drug with/without 3 *μ*M of HQ17(3) for 24 h. Cell viability was measured. Viability of untreated group is designated to be 100%. The experiments were conducted in triplicate. Means ± SD are shown.

Finally, we tried to elucidate any additive or synergistic effect when HQ17(3) is combined with other anticancer drugs. 5-FU, NaAsO_2_, and ABT-737 were tested. Leukemia cells Molt-4 and Ramos were treated with different doses of anticancer drug with or without 3 *μ*M of HQ17(3). The results are shown in Figure 5. Although synergistic effect was not found, an additive effect was prominent when HQ17(3) and the aforementioned anticancer drugs were used together. Specifically, HQ17(3) exerted no toxic effect on normal peripheral blood mononuclear cells at concentrations of up to 50 *μ*M [3]. In conclusion, the high sensitivity of leukemia cells to HQ17(3) may be associated with the reduction of topo II*α* and c-Myc activities, as well as with the downregulation of the miR-17-92 cluster expression. Natural product HQ17(3) itself may be an anticancer drug or have a significant function in sensitizing leukemia cells to anticancer drugs.

## Supplementary Material

U937 cells were cultured with/without 3 *μ*M of HQ17(3) for 24 h. The miRNA levels were detected by an Agilent human miRNA array R12. The raw data of microarray is presented here. For those miRNA levels shown apparent change (>1.43 fold or <0.7 fold, and signal >60) after HQ17(3) treatment, 4 of them were up-regulated (marked in red) and 21 of them are down-regulated (marked in blue).

## References

[1] Wu P-L, Lin S-B, Huang C-P, Chiou RY-Y (2002). Antioxidative and cytotoxic compounds extracted from the sap of Rhus succedanea. *Journal of Natural Products*.

[2] Lin TY, Huang CP, Au LC, Chang YW, Hu CY, Lin SB (2012). A cysteine-reactive alkyl hydroquinone modifies topoisomerase II*α*, enhances DNA breakage, and induces apoptosis in cancer cells. *Chemical Research in Toxicology*.

[3] Huang C, Fang W, Lin L (2008). Anticancer activity of botanical alkyl hydroquinones attributed to topoisomerase II poisoning. *Toxicology and Applied Pharmacology*.

[4] Nishida KM, Siomi MC (2006). Molecular mechanisms of RNA silencing by siRNA, miRNA and piRNA. *Tanpakushitsu Kakusan Koso*.

[5] Ghildiyal M, Zamore PD (2009). Small silencing RNAs: An expanding universe. *Nature Reviews Genetics*.

[6] Sheng XH, Du LX (2007). Progress on the research of microRNAs and its function in humans and animals. *Yi Chuan*.

[7] Wu W, Sun M, Zou G, Chen J (2007). MicroRNA and cancer: current status and prospective. *International Journal of Cancer*.

[8] Vardiman JW, Harris NL, Brunning RD (2002). The World Health Organization (WHO) classification of the myeloid neoplasms. *Blood*.

[9] Olive V, Jiang I, He L (2010). Mir-17–92, a cluster of miRNAs in the midst of the cancer network. *International Journal of Biochemistry and Cell Biology*.

[10] Zhang Z, An Y, Teng C (2009). The roles of miR-17-92 cluster in mammal development and tumorigenesis. *Yi Chuan*.

[11] Dews M, Homayouni A, Yu D (2006). Augmentation of tumor angiogenesis by a Myc-activated microRNA cluster. *Nature Genetics*.

[12] O'Donnell KA, Wentzel EA, Zeller KI, Dang CV, Mendell JT (2005). c-Myc-regulated microRNAs modulate E2F1 expression. *Nature*.

[13] Venturini L, Battmer K, Castoldi M (2007). Expression of the miR-17-92 polycistron in chronic myeloid leukemia (CML) CD34+ cells. *Blood*.

[14] Hong L, Lai M, Chen M (2010). The miR-17-92 cluster of microRNAs confers tumorigenicity by inhibiting oncogene-induced senescence. *Cancer Research*.

[15] Cloonan N, Brown MK, Steptoe AL (2008). The miR-17-5p microRNA is a key regulator of the G1/S phase cell cycle transition. *Genome Biology*.

[16] Olive V, Bennett MJ, Walker JC (2009). miR-19 is a key oncogenic component of mir-17–92. *Genes and Development*.

[17] Cao Y, Yu SL, Wang Y, Guo GY, Ding Q, An RH (2011). MicroRNA-dependent regulation of PTEN after arsenic trioxide treatment in bladder cancer cell line T24. *Tumor Biology*.

[18] Tao J, Wu D, Li P, Xu B, Lu Q, Zhang W (2012). microRNA-18a, a member of the oncogenic miR-17-92 cluster, targets Dicer and suppresses cell proliferation in bladder cancer T24 cells. *Molecular Medicine Reports*.

[19] Luo Z, Dai Y, zhang L (2013). miR-18a promotes malignant progression by impairing microRNA biogenesis in nasopharyngeal carcinoma. *Carcinogenesis*.

[20] Chen YR, Robin YC, Lin TY (2009). Identification of an alkylhydroquinone from rhus succedanea as an inhibitor of tyrosinase and melanogenesis. *Journal of Agricultural and Food Chemistry*.

[21] Geiss GK, Bumgarner RE, Birditt B (2008). Direct multiplexed measurement of gene expression with color-coded probe pairs. *Nature Biotechnology*.

